# Comparison of the analgesic efficacy of oral ketorolac versus intramuscular tramadol after third molar surgery: A parallel, double-blind, randomized, placebo-controlled clinical trial

**DOI:** 10.4317/medoral.21077

**Published:** 2016-07-31

**Authors:** Mario-Alberto Isiordia-Espinoza, Amaury Pozos-Guillen, Ricardo Martinez-Rider, Jose Perez-Urizar

**Affiliations:** 1 DDS, MSc, PhD. Departamento de Farmacología, Departamento de Posgrado e Investigación, Facultad de Odontología, Universidad Autónoma de Baja California, Mexicali, B.C., México; 2 DDS, MSc, PhD. Laboratorio de Ciencias Básicas, Facultad de Estomatología, Universidad Autónoma de San Luis Potosí, San Luis Potosí, S.L.P., México; 3 DDS. Departamento de Cirugía Oral y Maxilofacial, Facultad de Estomatología, Universidad Autónoma de San Luis Potosí, San Luis Potosí, S.L.P., México; 4 MSc, PhD. Departamento de Farmacología, Facultad de Ciencias Químicas, Universidad Autónoma de San Luis Potosí, San Luis Potosí, S.L.P., México

## Abstract

**Background:**

Preemptive analgesia is considered an alternative for treating the postsurgical pain of third molar removal. The aim of this study was to evaluate the preemptive analgesic efficacy of oral ketorolac versus intramuscular tramadol after a mandibular third molar surgery.

**Material and Methods:**

A parallel, double-blind, randomized, placebo-controlled clinical trial was carried out. Thirty patients were randomized into two treatment groups using a series of random numbers: Group A, oral ketorolac 10 mg plus intramuscular placebo (1 mL saline solution); or Group B, oral placebo (similar tablet to oral ketorolac) plus intramuscular tramadol 50 mg diluted in 1 mL saline solution. These treatments were given 30 min before the surgery. We evaluated the time of first analgesic rescue medication, pain intensity, total analgesic consumption and adverse effects.

**Results:**

Patients taking oral ketorolac had longer time of analgesic covering and less postoperative pain when compared with patients receiving intramuscular tramadol.

**Conclusions:**

According to the VAS and AUC results, this study suggests that 10 mg of oral ketorolac had superior analgesic effect than 50 mg of tramadol when administered before a mandibular third molar surgery.

**Key words:**Ketorolac, tramadol, third molar surgery, pain, preemptive analgesia.

## Introduction

Third molar surgery is the most common procedure carried out by oral and maxillofacial surgeons, and it is a common model for evaluating the efficacy of analgesics for acute dental pain relief (1). It is often associated with swelling, pain, and trismus (2). Pain associated with surgical removal of mandibular third molars ranges between moderate and severe during the first 24 hours (h) after surgery, with the major pain intensity occurring between 6 and 8 h when a conventional local anesthetic is used (3).

Preoperative administration of some analgesics has demonstrated reducing the onset of postoperative pain (1). It has been suggested that preemptive analgesia (analgesia given before the painful stimulus begins) is an alternative for treating the postsurgical pain of third molar removal (4). This therapeutic approach prevents or reduces the development of any “memory” of the pain stimulus in the nervous system. Preemptive analgesia may be defined as an antinociceptive treatment that prevents establishment of altered central processing of afferent inputs from injury sites. The most important conditions to achieve an effective preemptive analgesia are establish an appropriate blood level of analgesic before the surgical injury, and continuation of this effective analgesic level into the post-injury period to prevent central sensitization during the inflammatory phase (5).

Three classes of analgesic drugs have been used for pain control after mandibular third molar surgery: Local anesthetics, Non-Steroidal Anti-inflammatory Drugs (NSAIDs), and opioids (1,6-10). Ketorolac is an NSAID that has showed to be effective after oral and parenteral administration. This drug produces its effect through inhibiting synthesis of prostaglandins, the fatty acid that promotes pain (11-13). Additional mechanisms of action have been proposed to explain the efficacy and high potency of ketorolac, including a modulator effect on opioid receptors (14) and stimulation of nitric oxide release (11). Tramadol is an opioid analgesic effective in treating moderate to severe pain. It has a low addiction potential. It is used against multiple acute pain conditions, including postsurgical pain (15). It acts on opioid receptors and seems to modify the transmission of pain, inhibiting the reuptake of serotonin and noradrenaline (16). The aim of this study was to evaluate the preemptive analgesic efficacy of oral ketorolac versus intramuscular (IM) tramadol after a mandibular third molar surgery.

## Material and Methods

This pilot study was a parallel, double-blind, randomized, placebo-controlled clinical trial carried out in the Department of Oral and Maxillofacial Surgery of the Faculty of Dentistry, San Luis Potosi University, Mexico, following the guidelines suggested by the Consolidated Standards of Reporting Trials (CONSORT) group for planning and reporting clinical trials (17); conducted in accordance with the Declaration of Helsinki, and the Ethics Committee approved the study design. All the subjects were informed of the possible risks of oral surgery and experimental treatments, and they signed an institutionally approved consent form.

Inclusion criteria were age 18 to 25 years, either gender, free of systemic disease, clinical and radiographic diagnosis of impacted mandibular third molar, no pain associated with the subject third molar up to the day of surgery, and grade of surgical difficulty II, III or IV. Exclusion criteria were the use of analgesics 24 h before the procedure, history of seizure disorder, pregnancy or lactation, use of oral contraceptive, and known hypersensitivity to the study medications.

All subjects were informed of the possible risks of oral surgery and treatments used. Each patient accepted and signed an informed consent form. Patients were assigned sequential numbers in order of enrollment, and they received their allocated treatment according to a computer-generated randomization schedule prepared before the start of the study.

**Figure 1:**
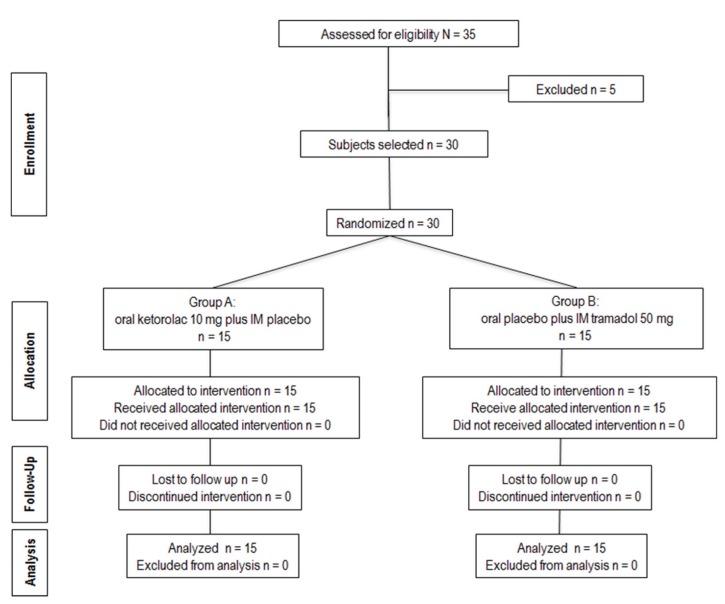
Clinical trial flowchart of a subject´s progress through the phases of the study.

Thirty patients were randomized into two treatment groups, each with 15 patients, using a series of random numbers: group A, oral ketorolac 10 mg plus IM placebo (1 mL saline solution); or group B, oral placebo (pill with physical characteristics similar to oral ketorolac) plus IM tramadol 50 mg diluted in 1 mL saline solution. These treatments were given 30 min before start the surgery. We evaluated the time of first analgesic rescue medication, pain intensity, and total analgesic consumption. The algorithm of the figure 1 shows the progress of subjects through the phases of the trial.

All surgical procedures were carried out in the Department of Oral Surgery by the same surgeon, and evaluations were carried out by an independent investigator. Anesthesia was by nerve block of the lingual, buccal, and inferior alveolar nerves using two 1.8-mL cartridges of 2% lidocaine containing 1:100,000 epinephrine. Once anesthesia was obtained, surgery was started. A mucoperiosteal flap was prepared by making an incision distal to the mandibular second molar along the anterior edge of the ascending ramus of the mandible (this flap was used to close the surgical wound). Suturing was done with 4-0 silk, and the number of sutures was documented. Mandibular third molars were classified according to the Winter Classification, in addition to the Pell and Gregory classification. Surgical difficulty was based on a modified scale of Parant, as follows: grade I, extraction with forceps and elevators; grade II, extraction by osteotomy; grade III, extraction by osteotomy and coronal section; grade IV, extraction by osteotomy, root, and coronal section (18). In all cases, duration of the operation (from incision to final suture) was recorded. In each patient, a partial bony impacted mandibular third molar was extracted.

A 100-mm visual analog scale (VAS) was used to assess pain. The VAS consisted of an interval scale ranging from 0, representing no pain or discomfort, to 100, representing maximum pain or discomfort. The VAS report was recorded each hour for 8 h after completion of surgery, and a last evaluation was done at 24 h post-surgery. Patients were given four oral ketorolac 10 mg pills and were instructed to take one pill for rescue medication at least 6 h apart, according to their requirements. At the end of the evaluation period (24 h), patients returned the unused oral ketorolac. Pills were counted to determine the number of consumed pills and patients in each group not needing any pills. Those patients having no pain relief 30 min after taking ketorolac were given sublingual ketorolac 30 mg as a rescue analgesic procedure. Total analgesic consumption (oral and sublingual ketorolac) was evaluated.

Patients were asked to provide an overall evaluation of the analgesic efficacy according to a three-point categorical scale, at the end of the trial. The categories of scale were 1: poor (lots of pain), 2: fair, 3: good (minimum pain). Both patients and the independent evaluator were blinded regarding the administered treatment. Intra- and postoperative complications and adverse events were recorded.

Power analysis of this pilot study was performed using the data from the VAS in the third postoperative hour as the response variable. The expected difference in means was 27.66 mm; while the expected standard deviation was 20.95 mm; including 15 patients in each treatment group and an alpha value of 0.05. Taking into consideration the data of the response variable, the power of this study was 93%.

Data are expressed as mean and standard deviation, median and ranges or number of frequency and percentage. The Fisher exact test was used for to evaluate the sex, surgical difficulty, patients without needing analgesic during the period of evaluation (8 h) and patients requiring rescue analgesic procedure (sublingual ketorolac). For analyze the age, weight, duration of surgery, number of suture, total analgesic consumption, and the VAS scores the Student t test was utilized. The Mann-Whitney U test was used to evaluate the time of first rescue analgesic and the overall evaluation of analgesic treatments. The area under the curve (AUC) of the VAS was calculated using the trapezoidal method and was used to evaluate the overall pain intensity. A *P* value less than 0.05 was considered significant statistical difference.

## Results

**Figure 2:**
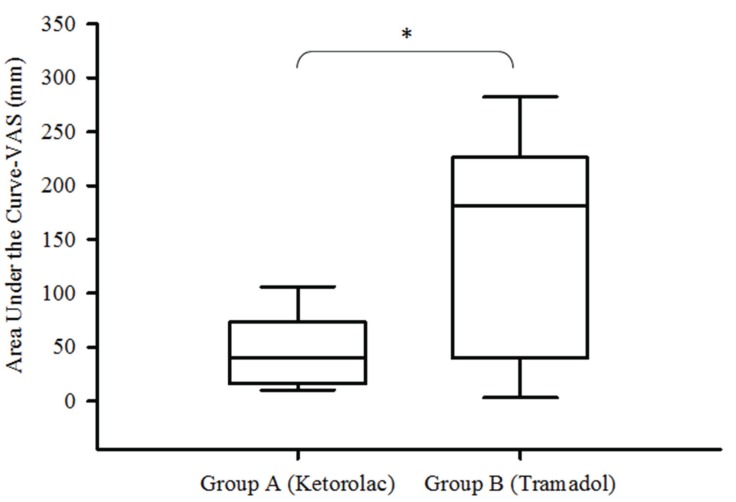
Pain intensity evaluated by AUC of VAS. Group A, oral ketorolac 10 mg plus intramuscular placebo; Group B, oral placebo plus intramuscular tramadol 50 mg (*P*=0.043).

A total of 35 patients were enrolled in the study of which 30 patients were included in this clinical trial. The statistical analysis was done using the data from all included patients (Fig. 1). Personal and surgical variables were similar among the groups ( Table 1). The time of first rescue analgesic medication was longer in the patients taking oral ketorolac when compared with patients receiving IM tramadol (*P* = 0.01). In the same way, the patients of group A had less pain when compared with patients of group B, the AUC of VAS showed statistical difference (*P* = 0.04; Fig. 2). However; others indicators of analgesic efficacy evaluated through the study - patients not needing analgesic during the period of evaluation, patients requiring rescue analgesic (sublingual ketorolac 30 mg), and analgesic consumption - did not show statistically significant differences among the treatment groups ( Table 2).

**Table 1:**
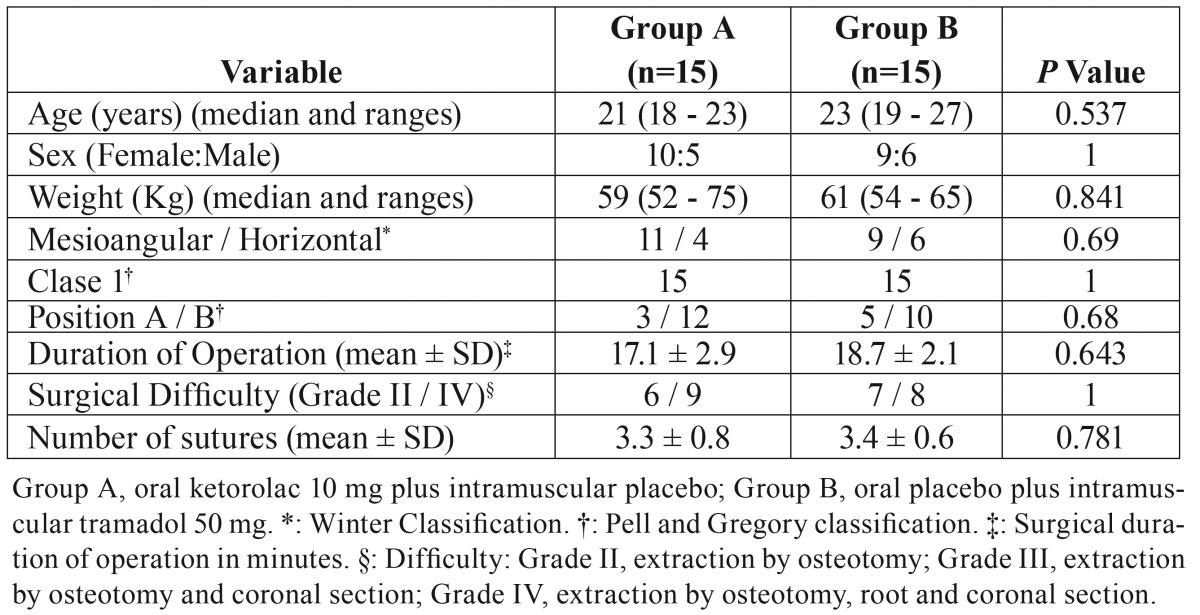
Personal and surgical data.

**Table 2:**
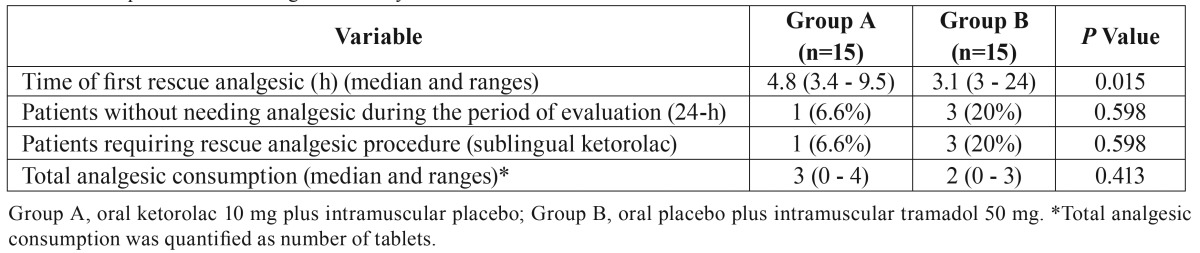
Comparison of the analgesic efficacy.

**Figure 3:**
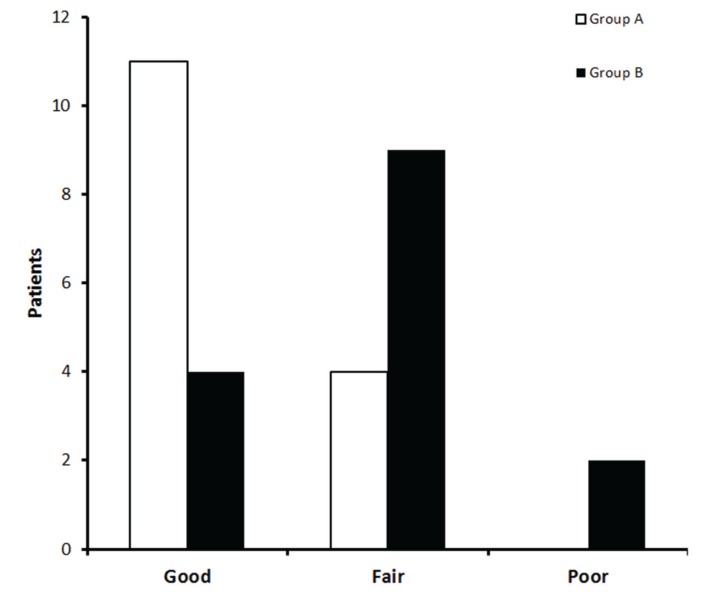
Overall evaluation of analgesic treatments. Group A, oral ketorolac 10 mg plus intramuscular placebo; Group B, oral placebo plus intramuscular tramadol 50 mg (*P*=0.027).

Patients taking 10 mg of oral ketorolac plus intramuscular placebo presented lower pain intensity scores in the third and fourth post-operative hour according to VAS when compared with patients who receiving oral placebo and 50 mg of intramuscular placebo ( Table 3). Moreover, the distribution of scores of overall evaluation of the analgesic treatments showed more patients in the ketorolac group (73.3%) who informing a good effect of analgesia when compared with tramadol group (26.6%) (*P* = 0.02; Fig. 3). There were no complications associated with the surgical procedure itself. No patient reported adverse events.

**Table 3:**
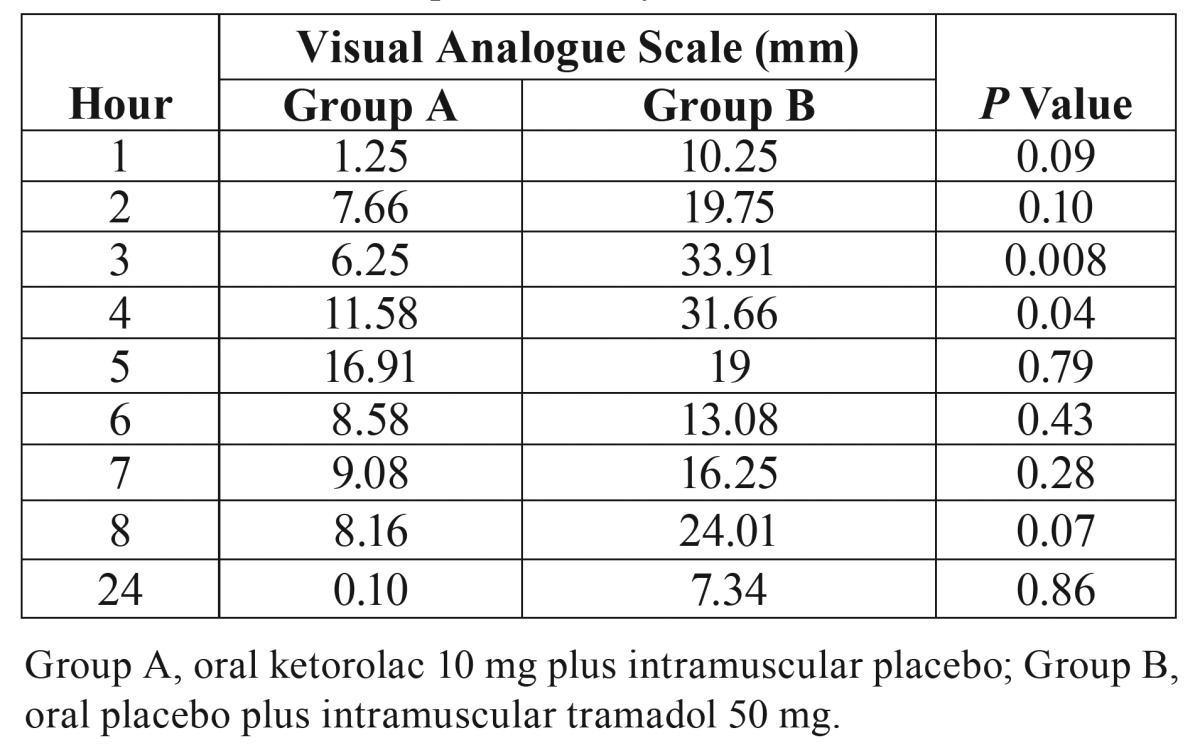
Assessment of pain intensity.

## Discussion

The results of this study shown that patients who took 10 mg of oral ketorolac had an improved analgesic efficacy according to the VAS scores as well as with the AUC of VAS, and a longer time of first rescue analgesic medication than those patients receiving 50 mg of intramuscular tramadol. Few studies have compared tramadol and ketorolac for the pain relief after oral and maxillofacial surgery (19-22). Gopalraju *et al.*, (19) compared the intravenous analgesia of 30 mg of ketorolac and 50 mg of tramadol after third molar surgery, proving ketorolac to produce better control of pain. Mishra *et al.*, (20) carried out a double-blind, randomized, clinical trial evaluating the analgesic efficacy of both drug and concluded that the postoperative administration of 100 mg of tramadol is as effective as 20 mg of ketorolac in the relief of pain. Shah *et al.*, (21) and Ong *et al.*, (22) made two separate clinical studies to assess the preemptive analgesic effectiveness of 30 mg of ketorolac and 50 mg of tramadol using intramuscular and intravenous administration respectively, and informed that ketorolac is better than tramadol for management of pain prior to oral surgery. Moore *et al.*, (23) executed a clinical trial evaluating the analgesia of dexketoprofen-tramadol combination in acute dental pain. This study included 2 treatment groups with individual tramadol (37.5 and 75 mg), 2 groups using dexketoprofen trometamol (12.5 and 25 mg), and an ibuprofen group (400 mg). The results show that both dexketoprofen trometamol and ibuprofen produced a better analgesic effect when compared to tramadol after third molar surgery. In addition, the data confirms that tramadol has an inferior analgesia in this kind of surgical procedure.

The results of this clinical trial showed that the ketorolac group had a longer period for the consumption of the first analgesic tablet after surgery when compared to the tramadol group. The data shows that 1 patient in the ketorolac group and 3 of the tramadol group did not require an analgesic during the period of evaluation. The trends of outcomes of this variable are confusing in relation to the analgesia observed for ketorolac and tramadol in this study, which could be explained by the personal variability and the subjectivity of the measurement of this variable by patient demand. The same number of patients was obtained for whom requiring rescue analgesic medication. This is in agreement with other variables in this clinical trial. It is important to note that these variables evaluating the analgesic efficacy did not show statistical difference.

The superior analgesic effectiveness of ketorolac in comparison to tramadol could be explained by the pathogenesis of dental pain, which is largely inflammatory. The evidence-based medicine has shown that NSAIDs are the best analgesics for dental pain treatment (4). It is possible that higher doses of tramadol have a better analgesic effect than ketorolac. However, the incidence of side effects, particularly nausea and vomiting, may be high. Drowsiness, nausea, and dizziness are the most common adverse effects of tramadol (15,16). In a study of the effects of tramadol on dentoalveolar surgical pain by Collins *et al.*, (24), 39% of patients on high doses of tramadol (100 mg 4 times a day orally), 12% on moderate doses (50 mg 4 times a day), and 6% on low doses (50 mg twice a day) withdrew from the study owing to nausea, vomiting, dizziness, or drowsiness. Interestingly, Pozos-Guillén *et al.*, (25) used high doses of pre- and postoperative tramadol (100 mg) but the analgesic effect was similar to that of what we found in this study using 50 mg of tramadol; which indicates a limited analgesic effect of tramadol, even while using high doses after third molar surgery. Moreover, a systematic review and meta-analysis demonstrated that a single dose of tramadol has a significantly inferior analgesic effectiveness and safety profile than NSAIDs in oral surgery (26).

Effective and safe analgesia is one of the many challenges in health cares. The preoperative use of ketorolac is not very common due to its inhibition of prostaglandin I2 and thromboxane A2 synthesis, which may cause peri- and post-operative bleeding (27). In this study no patients reported bleeding complications. In this sense, some clinical studies have used preoperative ketorolac by different administration-pathway in oral surgery without reporting bleeding complications (24,28,29).

In this clinical trial five patients were excluded. These patients did not participate in the study because 2 patients presented pain associated with the subject third molar up to the day of surgery, 2 patients took analgesics a day before surgery, and 1 patient was allergic to NSAIDs.

According to the VAS and AUC results, this study suggest that 10 mg of oral ketorolac had better analgesic effect than 50 mg of tramadol when administered before a mandibular third molar surgery. Further studies employing a larger sample size are necessary to confirm these finding.
